# Nutrition Justice: Uncovering Invisible Pathways to Malnutrition

**DOI:** 10.3389/fendo.2020.00150

**Published:** 2020-03-24

**Authors:** Sarah Hanieh, Holly High, John Boulton

**Affiliations:** ^1^Department of Medicine, Peter Doherty Institute of Immunity and Infection, University of Melbourne, Melbourne, VIC, Australia; ^2^The Department of Anthropology, Faculty of Arts and Social Sciences, The University of Sydney, Sydney, NSW, Australia; ^3^School of Medicine and Public Health, University of Newcastle, Newcastle, NSW, Australia

**Keywords:** nutrition, malnutrition, child, adaptation, food security, hunger, justice

## Abstract

We propose the use of the analytic frame of “nutrition justice” to reconcile the separate imperatives of Global Health for nutritional sufficiency for all, the requirement to eradicate childhood malnutrition, and the need for strategies to check the emerging pandemic of the double burden of malnutrition in the Global South. Malnutrition and its consequences of growth stunting are the result of disruption to the nutritional ecology of childhood from structural violence. This is mediated through loss of food security and perturbation to the cultural status of food, and on the prerequisites for nurture during infancy and early childhood. These socio-political factors obscure the role of biological adaptation to nutritional constraint on growth and hence the causal pathway to the double burden of malnutrition. In this paper we describe how the effects of historical and contemporary structural violence on the nutritional ecology of childhood are mediated using the examples of remote Aboriginal Australia and the Lao PDR. Both populations live by force of circumstance in a “metabolic ghetto” that has disrupted the prerequisites for parental nurturing through loss of food security and of traditional sources of transitional staple foods for weaning. Growth faltering and stunting of stature are markers of adaptation to nutritional constraint yet are also the first steps on the track to the double burden. We discuss the implications of these observations for strategies for global food sufficiency by mean of a thought-experiment of the effect of food and nutrient sufficiency for growth on future health and metabolic adaptation.

## Introduction

The ability of our speciesto adapt to almost all of Earth's environmental niches is the hallmark of the success of our survival strategies, but contains the seeds of its demise in the face of the inexorable increase in the global population, the persistent wastage of food, and from the impending effects on climate change on water and food security. Climate change is considered to be the greatest global threat to health for the twenty-first century with the poorest nations most at risk adverse effects. The world population is projected to reach 9 billion or more in the next 30 years, with most of the growth occurring in the poorest nations (1, 2). These have the least capacity for either adaptation or mitigation and will suffer most from the complex but fixed relation between future population growth and the adverse effects on health (3).

The key to human adaptation is hidden in the critical period during the transition from the separate endocrine mediators of fetal and postnatal growth. Karlberg's Infancy-Child-Puberty model shows how and when these three phases of growth interact to create the characteristic shape of the child's growth curve over time (4). A marked delay in the time of transition from the Infancy to the Childhood phase results in a reduction of final stature below the genetic potential of the individual (5–7). However, the deep factors that influence the timing of the transition from the Infancy to the Childhood phase that occurs from 9 months onwards as the fetal influences on growth ebb, and which provide the ability to slow growth in response to nutritional constraint, are typically obscured by the cultural context. This is because the individual child either suffers malnutrition and needs urgent nutritional rehabilitation within the context of humanitarian health intervention, or because the child with growth faltering lives in a community in a subsistence economy and is unremarkable due to the normalization of morbidity. In both situations the complexity of the causal pathway remains obscure to the health professionals involved because they extend beyond social determinants that typically limit the extent of the public health model (8).

We emphasize that this perspective interrogates qualitatively different factors on the causal pathway to childhood under-nutrition, growth faltering and stunting, and runs in parallel to, and complements, those that have been systematically documented over the past six decades. These extend from the effect of the allostatic load from the metabolic burden on the immune system from recurrent respiratory and gastro-intestinal infection with impaired small bowel absorptive capacity, that is in turn exacerbated by an unhygienic environment; and from barriers to food security (9), within social marginalization, poverty, disempowerment, and structural violence (10).

The ability to respond to the nutritional environment through a change in the tempo of growth in early development offers a literal and metaphorical key to addressing both sides of the coin of the challenge of global food security. For post-industrial high-income nations a shift in food production and use is necessary for ecological reasons; for emerging economies (low-income nations), erasing the double burden of malnutrition is the pressing challenge. The need to encompass a wide-angle view is expressed by the calls for “A new nutrition manifesto for a new nutritional reality”(11) and also “A future direction for tackling malnutrition” (12). The term “nutrition justice” has previously been used by Wells (13), Swinburn et al. (14), and Swinburn (15) and highlights the moral imperative for equity in food security, and hence builds on the origins of the deep causes of the global syndemic of obesity and malnutrition as being within structural inequity (16), as well as food as the commodity that has been at the base of the pyramid of power since before the Neolithic farming revolution (13).

In this paper we argue that these apparently categorically separate problems are inextricably linked when viewed from the perspective of what we call “nutrition justice.” Although previous evidence has demonstrated a link between malnutrition and global inequity (17, 18), we use the expression “nutrition justice” to highlight that the prerequisite for global food security goes beyond the humanitarian approach to the child suffering malnutrition as a victim of social marginalization, poverty, and conflict, and that it should include an analysis of how the invisible forces of structural violence operate to embed such disadvantage and prevent equity in access to optimal nutrition.

In this paper we propose that the concept of childhood adaptation to nutritional constraint with a change in the tempo of growth at a critical period, expressed as phenotypic plasticity, could be used as a model beyond the niche of evolutionary developmental biology, and specifically that it could provide an explanatory model for the contemporary challenges of global nutrition. The first of these is the problem of childhood malnutrition, with its consequence of growth faltering and loss of final height potential, which leads to stunting of stature. The second is the imperative for global food security as Earth's population heads toward 10 billion people: a 10-fold increase in 250 years (19).

We argue for the use of the concept of adaptation to achieve an explicit connection between the intractable problem of childhood malnutrition in marginalized communities and the moral challenge for the world as a whole to achieve a sustained level of food production sufficient to ensure global food security. In this context we use adaptation both in its literal sense in respect of the fall in tempo of growth to nutritional constraint, and in its metaphorical sense in terms of the radical changes that are required to achieve global food security.

The expression nutrition justice was coined during discussion between the authors on the paradox of endemic growth faltering from malnutrition in the midst of plenty amongst children in communities in remote Aboriginal Australia (8). These children grow up in a metabolic ghetto within a wealthy nation (13), suffering endemic growth faltering during the first year of life. They go on to carry a high risk of the early-onset of metabolic syndrome with its life-shortening consequences (20, 21). Growth faltering amongst Aboriginal children goes beyond being an irreversible health problem into the moral dimension of children's rights. This intersects with a separate question, which is: How can 10 billion people be provided with sufficient food to allow each child to reach her growth potential?

At present the focus of the EAT-Lancet Commission is on how post-industrial high-income nations should respond with respect to the achievement of the structural changes in the mechanisms of food production and through ecosystem changes (19). This top-down approach detracts attention from the question of how the eradication of childhood malnutrition is to be achieved. If the broad-brush aim of the Commission is that people in the global North should eat less, or less of some food groups, so that more is available to the global South, then it begs the questions of how much more food energy is needed for each child at the microcosm of the village in Nepal, Indonesia, Vanuatu, or Laos, and how will that be achieved at the village, district, and national levels?

Our aim is to close the conceptual space using an evolutionary-developmental approach that starts with a thought experiment: since nutrition justice requires that all babies and children should be fed sufficient food energy for optimal growth, then what are the practical implications for the achievement of the necessary additional food energy and protein intake?

## Adaptation

The notion that growth faltering represents an adaptive phenomenon with its onset situated at a critical period of somatic development (and seen as a delay in the time of transition from the Infancy to the Childhood phase that results in a reduction of final stature) (5–7), rather than a perturbation of homeostasis that can be normalized with medical intervention, requires a shift toward the interdisciplinary perspective of evolutionary developmental biology.

At the end of the Pleistocene and the beginning of the Holocene, c 12,000 years before present (B.P.) societies shifted from hunter-gatherer toward a mixed food economy in the Near East. The Neolithic farming revolution took place in several separate sites more or less simultaneously (22). With technological changes in food production came changes in social relations. Importantly, food became a resource that could be stored, hoarded, and controlled. Even before the Neolithic Demographic Transition (23, 24), there is the first evidence of food being used to mediate political power (13). Archaeological evidence from the Natufian culture suggests rapid climate change during the Younger Dryas stadial (11,700–10,500 years B.P.) was a critical factor in this radical change. Climate and food production changed faster than evolutionary adaptation in human populations, and this was reflected in the marked fluctuations in population in this era (25, 26). From this perspective, the success of hominins is an outcome of the enhanced evolved capacity for metabolic adaptation within an individual's lifetime or between generations to different ecological zones with an unpredictable food supply (10). There is plausible evidence that this capacity emerged amongst our hominin antecessors in response to rapid changes in the environment from climatic fluctuations during the upper Pleistocene (27).

Wells characterizes as a “metabolic ghetto” the populations of, or within, a nation that demonstrate such phenotypic adaptations (13). These phenotypic adaptations include lower muscle bulk, shorter stature, and a metabolism adapted to parsimony. These characteristics stem from undernourishment in fetal life or early childhood, leading to structural and functional changes in organs responsible for nutrient regulation (e.g., adipose tissue and pancreatic beta cell dysfunction). In conditions of food abundance in post-natal life this may lead to preferential deposition of visceral (central) adipose tissue and insulin resistance and predispose the child to development of obesity and Type II diabetes (28).

At a population level, nutritional constraints over successive generations leads to the selection of phenotypic traits that represent trade-offs between survival and thriving. The double burden of malnutrition with early morbidity from diabetes and cardiovascular disease is the cost of adaptation in previous generations. Laboratory studies provide a glimpse of epigenetics mechanisms which may be relevant in humans (29, 30).

The metabolic ghetto aptly describes the situation in remote Aboriginal Australia. The fine-tuned adaptation to the nutritional environment of pre-contact society was violently disrupted and replaced by a limited range of foods supplied as rations within a clear hierarchy of power (31), which was followed by documented evidence of transgenerational malnutrition (32). It is in this context that the high rate of heart disease and diabetes amongst such populations should be understood. We argue that these are not “lifestyle” diseases as such, but are the result of nutritional constraint over both the long and short term. Since colonialism, the evidence is clear that malnutrition in this case has been closely linked to questions of power and marginalization.

In Australia the double burden affects two distinct population groups, Aboriginal people and South Asian migrants (33, 34). The Aboriginal population in Australia carry a high burden of obesity from late childhood, and a high prevalence of early-onset metabolic syndrome with onset of insulin-insensitive diabetes in adolescence. The high risk of cardiovascular disease in both Aboriginal and South Asian populations is in marked contrast to the rapid rate of decline in mainstream society with the death rate from cardiovascular disease in mainstream Australian population falling by 80% in the past four decades. For example, in the age group 75–79, the rate fell from 125 to 25 per 100,000 per year (35), whereas amongst Aboriginal men aged in the 55–74 age group, nearly half were at high risk for CVD, and 20–26% had known symptomatic heart disease (33).

West-Eberhard provides an evolutionary interpretation to the double burden of malnutrition: childhood malnutrition followed by the early-onset onset of morbidity from obesity and metabolic syndrome (36). In putting forward the “visceral adipose tissue prioritization” hypothesis she argues that “socially subordinate individuals are under selection to adjust to the consequences of limited resources … including alternative traits that salvage elements of their compromised survival and reproduction,” and contrasts these effects with the traits selected in the socially higher ranked individuals (“who were more involved in high-stakes social competition and less exposed to hunger and infection”) for subcutaneous adipose tissue and stature, amongst other morphological features that signal higher value for reproductive success. She argues that this reflects selection bias from the social stratification that emerged within agricultural societies and that prioritization of deposition of visceral adipose tissues reflects a local response to the metabolic load from immune stimulation from the increased burden of gastrointestinal infection and infestation from poor sanitation in sedentary groups, and its adverse effect on nutritional status.

Strategies to eradicate the double burden of malnutrition require a shift in perspective in order to frame its etiology as the consequence of a metabolic maladaptive response to long-standing population adaptation to nutritional constraint produced by a particular power arrangement. This raises the question of whether it is possible to reconcile the need for the improvement of childhood nutrition in a population in which growth faltering and stunting is endemic when there is strong evidence that the shift to a diet high in refined carbohydrate and fat results in metabolic maladaptation, and is the leading etiological factor to the double burden of malnutrition (37, 38).

## Structural Violence

The emergence of the double burden of malnutrition and syndemic of obesity and diabetes highlights the cost of disruption to adaptation of childhood growth to transgenerational nutritional constraint. However, its distribution uncovers evidence for the “known unknown” structural factors that act as barriers to the normalization of homeostasis. From a Western scientist's perspective it is clear that the double burden is emblematic of the transition from a pre-industrial to a post-industrial economy, and the disruption of transgenerational adaptation of fetal and childhood growth amongst those ranked lowly within the global economy. A second-order consequence of this burden of morbidity is on its deleterious effect on the rate of increase of life expectancy over the past decades for those aged over 60 years in low and mid-income countries, which has risen at only half the rate of that in high-income nations (39).

The concept of the metabolic ghetto provides an explanatory model for understanding the transgenerational effects on metabolism: evolving from an adaptation to being trapped by the forces of power in an adverse nutritional environment, to a transition to food security or access to high quality food. The effects of such a situation are best described by the term structural violence. This is violence that is evident on bodies, psyches, social relations, and health not through an overt act of might, but indirectly as an effect of a system of social relations such as political, legal, and economic arrangements. For instance, it is evident in the physical, intergenerational effects of discriminatory legislation. It is evident in the health conditions and social disintegration of marginal populations where social gradients and wealth are steepest, and where racism is ingrained. The term structural violence was coined by the Norwegian peace philosopher Galtung (40), and has since been popularized by the American public health physician Paul Farmer who has emphasized the damaging trans-generational effects of structural violence on both personal agency and on the individual's physical and mental health (41).

The causal pathways of structural violence and its effects are most apparent when examined among a distinct group within a population. Australian Aboriginal people provide a tragic example because they were victims of legal, physical, territorial, and social exclusion from the time of British settlement in the late eighteenth century. This became entrenched through legislation from the 1880s onwards in each jurisdiction. For instance, Aboriginal children were excluded from school, families were excluded from their land which was a source of nutrition; and men were excluded from unions so that they could not get paid work through until the mid-twentieth century (42). For some, as clients of today's byzantine and punitive welfare state, the effects of structural violence can be understood as a colonization of the mind that exerts a profoundly debilitating effect on personal agency (43).

When the colonial frontier zone extended to the tropical savanna region of northern Australia and vast cattle stations took over the country in the late nineteenth century, the population of Aboriginal people collapsed as a consequence of massacres, influenza epidemics, and hunger (44–46). The remnant populations worked on cattle stations as an unpaid workforce under conditions of dire deprivation and hunger (32, 45). The legacy of transgenerational malnutrition is now evident in the endemic rate of non-communicable disease from metabolic syndrome (20, 38). The adverse effect on fetal and early postnatal growth is compounded by a complex mix of cultural factors in regard to the hierarchy of food items and choices (47), and adverse health behaviors, particularly smoking, and alcohol consumption during pregnancy that affect birth weight (48–50). For instance, how food is used within the family is a crucial question for nutrition. This was illustrated by the ethnographic method used in separate studies in Trinidad and Nauru that delineated the depth of inequality from structural violence from historical that accounted for the extent of NCD morbidity (51).

Structural violence is also perpetrated through invisible conflicts between the deep patterns of behavior of mainstream Western society and Aboriginal society. This can be seen in the collision over cultural attitudes and value-beliefs about feeding babies during the critical growth phase of the transition from breast-feeding to a mixed diet, and how it has adverse effects on food energy intake and hence growth faltering. Families two or three generations distant from their pre-modern hunter gatherer ancestors do not share the same belief in the equation that has been internalized over millennia in families of the descendants of Neolithic European farmers and gardeners that food intake is a necessary investment in a child's growth, stature and strength, and hence their future capacity as a farmer or warrior (8). Hunter-gatherers had ample access to food resources, so skill as a hunter, story-teller, and in ritual expertise were valued above brawn. A peripatetic lifestyle meant that mothers carried their babies, so a heavy baby was a disadvantage. Aboriginal grandmothers now do not wish to understand children's nurses and doctors' fixation on weight and growth.

Babies in Aboriginal society were accorded autonomy as a sentient individual (52), and were (and still are) expected to make choices in a way that is in conflict with the pattern of heteronomous parenting in which the parent decides when and how the child should eat, sleep, and wear (8). The result is that babies and toddlers were offered the breast and finger-food, but it was (and is) their decision as to whether they would eat. Anorexia after an illness therefore leads to growth faltering, as shown by figures from the public health Healthy Under-5s Kids program of the Northern Territory where one-fifth of children are under-weight or under–height for age (53).

Yet these factors are often invisible to public health approaches. The Western public health approach takes an immediate approach. It addresses problems as they surface and become apparent in clinics and emergency departments. This obscures the evidence for the effects of entrenched structural inequalities and the transgenerational consequences of morbidity from maladaptation, as well as from the deep cultural factors.

The effects of structural violence are also apparent in Western social democracies. For example in the UK data from the Biobank study shows that inequality is expressed through phenotypic and economic disadvantage. Men of lower social status are shorter, and women are fatter (higher BMI), with BMI having an inverse relation with income (54).

## Global Food Security

At present nearly one billion people are hungry, two billion people do not have regular access to sufficient food, and the number of chronically undernourished people increased by 10 million between 2017 and 2019 to 821 million (16). Childhood malnutrition accounts for nearly half of child deaths under the age of 5 years (11). Yet two billion people eat too much and of the wrong food (55). Although the global average daily energy intake has increased over the past three decades to 2,710 Kcal, the poorest nations have seen the smallest increases (+80 Kcal), and the lowest increase in protein (56). The second Sustainable Development Goal is to end hunger, achieve food security and improved nutrition, and promote sustainable agriculture (14, 19). The “EAT-Lancet Commission on health diets from sustainable food systems” advocates global transformation of the food system that would allow food for 10 billion people (19).

For SDG 2 to be met (57) with respect to the eradication of hunger, a reciprocal response from the global North is required. This includes an international commitment to a healthy diet in the global north: reduced meat consumption; an increased intake in legumes, fruit, nuts, and vegetables; the re-orientation of agriculture; sustainable food production; coordination of government of land and oceans; and halving of food waste (55). Since the geopolitical changes of globalization and poverty affect food security, trade policies need to be reformed to facilitate food security, and sustainable food systems (58).

Climate change threatens the conditions for the human needs for food, water, sanitation, and shelter to be met (1) The most severely affected will be the 2.5 billion farmers, herders, forest-dependent, and fishermen who depend on renewable resources (59). The effect of climate change on food production and security in low-income nations has widespread implications with regards to the increase in childhood malnutrition, with adaptation through growth faltering and stunting of stature, followed in adult life by the burden of non-communicable disease. For example in West and Central Africa under-nutrition increased from 33.5% in 1990–92 to 41.3% in 2014–16, and stunting in Africa will increase by 7% by 2030 (1). The OECD predicts that food availability will fall by 2025 by 3.2% due to climate change and worsening morbidity due to infectious disease so that there will then be a food deficit in most low-income countries. Climate change will affect all, but by 2030 the effect of climate change and poverty will severely disrupt the lives of 35–122 million marginalized people (1).

In a global survey of the prevalence of the double burden, when defined using the score for the Global Hunger Index (a combination of three indicators of mortality in early childhood and morbidity from malnutrition) of 20 or greater (60), and the prevalence of obesity, with a BMI of 30 kg/m^3^, of 15% or more, Iraq, Guatemala, Namibia, Lesotho, Swaziland, and Botswana were identified (60). This highlights the challenge of human adaptation in its widest sense to the emergence of a global syndemic of obesity, under-nutrition, and climate change (14).

The concept of nutrition justice requires sufficient food available for the world population and that this is contingent on its equitable distribution even without pressure on food systems in relation to the population. The emphasis of the place of children in the ecology of global nutrition and within the current discourse on the future for global nutrition highlights the need to reconcile separate factors that are in evident opposition. The humanitarian approach to intervention for childhood malnutrition in nations in which malnutrition is endemic (61, 62), therefore contains an internal paradox: that if all children were to be provided with sufficient food to achieve their genetic potential for height then there would not be sufficient food available within that nation. This highlights the gap between the clinician's short-term strategy of nutritional rehabilitation and the need to consider the structural and cultural consequences for children in countries (such as Laos which is the focus of the thought experiment that illustrates this point), in which the phenotypic adaptation of short stature allows homeostasis with the available food supply within a self-sufficient subsistence economy (63).

The question is therefore how to reconcile the two conflicting imperatives of optimal health for all with nutritional sufficiency, and yet equitable distribution of food resources. This question is dealt with tangentially by The Lancet Commission (19). This Commission advocates for global food sufficiency for a world population of 10 billion people through a radical alteration of strategies for food production and the type and quality of food raises several questions. Recommendations include obtainment of protein sources primarily from plants, doubling of the consumption of fruits, vegetables, legumes, and nuts; and more than 50% reduction in global consumption of red meat and foods with added sugars.

The first question is how this can be achieved in the face of evidence for an increase in the discrepancy between food production and consumption in several countries in Africa, particularly those in which is there is no evidence of a reduction in the rate of increase in the population.

The second question is whether global food energy sufficiency can be achieved with the recommended strategies and yet be sufficient in protein and micronutrient intake to provide equity of opportunity for every child to achieve their genetic potential in height. This would be contingent on a major shift in food resources in countries, particularly in South and S. East Asia, which are now food sufficient but at the cost of transgenerational metabolic adaptation to a nutritional plane provided by a low-protein rice or vegetarian-based diet.

## A Thought Experiment

Our notion of nutrition justice requires that every child has sufficient food to reach her genetic potential for height. If this were achieved, there would be no difference in mean stature between countries because of nutritional constrain. In this thought experiment we seek to identify the cultural, economic, and logistic barriers to this nutritional utopia using as an example the hill village of Kandon in north-east Laos in which author Holly has conducted ethnographic field studies over many years (63). Our starting point is the question: “if every child in the village aged from 6 to 18 months had one egg per day, and every child from 18 months to age 5 years had 300–500 Kcal more than she does now, where would the food come from, and what would the effect be at a district level?” The reason for using the addition of a daily egg to the diet is because it is a realistic food source in that village, and because it has been shown that children aged 6–9 months assigned one egg per day for 9 months showed increased height and weight for age, although its benefit was not maintained at 24 months in the absence of that additional source of food energy (64, 65).

In Kandon village there are 1,260 individuals, of whom 13 are children aged 6–18 months. If each were given one egg a day in additional to their usual diet, that would require that the village source nearly 5,000 extra eggs a year. The village currently has 1,007 individual birds. Assuming 15% (151 individual birds) are roosters, and 20% (201 individual birds) are immature, this leaves 655 hens at most. The average hen lays 63–100 eggs a year. So, the village would produce between 41,265 and 65,500 eggs a year. This works out at 33–52 eggs per individual man, woman and child in the village per year. That is, the existing egg production in the village is not sufficient for one egg per person per day if the eggs are spread evenly.

In addition, there are serious questions over whether eggs already produced by village chickens are routinely used for consumption. In observations in the village, eggs from village chickens did not form an important part of the diet in this village and are mainly used for hatching. In an intensive study of the diets of this and other ethnic Katu villages in the same District, a nutritionist made an extensive report of what people ate, and did not mention eggs a single time in her 245-page report (66). It was furthermore my observation, and this was confirmed in interviews, that poultry mortality was high. In at least one case, a mass mortality of poultry in the village was confirmed through laboratory testing as Highly Pathogenic Avian Influenza H5N1 (67). This study suggested that HPA H5N1 spills over from commercial chicken farms abroad and then travels to rural Laos via trade routes, where it infects and kills village chickens before dying out there, only to be reintroduced through the market once again. Studies of village chickens in low income settings have noted that “In situations where mortality rates are high, village poultry eggs are rarely consumed as they are prioritized for hatching” (68, 69).

Giving one extra egg to children aged 6–18 months to consume would represent a major diversion of eggs away from current uses for sustaining the poultry population of the village toward using eggs for consumption. The best means to achieve this would be to introduce vaccinations for common poultry illnesses, especially Newcastle Disease (ND). ND is very common, can be lethal to chickens, and furthermore presents with signs that are indistinguishable from HPA H5N1. A vaccination program would have the dual benefit of reducing the impact of ND and increasing the likelihood and effectiveness of H5N1 reporting, which is currently very rudimentary in Laos (67).

In the absence of such a program, it seems inevitable that if people were incentivized to buy an egg a day for each child, for instance by introducing a subsidy for this purpose, eggs for consumption will be sourced instead from the market. The closest market is 40 min away by motorcycle. However, there is a consumer preference in this village to know where food comes from and avoid market food. The eggs in the market usually come from Thailand and Vietnam, but their exact provenance is unknown and the security of the cold chain is uncertain. It is an unregulated market with regard to how the chickens were fed and how old the eggs are, and the eggs are a known source of Salmonella. Food available for sale in the market is also associated with heavy use of pesticides, insecticides, and various drugs (what is glossed as *khemii* in Lao, meaning simply “chemicals”). People also understand that the market is a source of infection, such as what we know as H5N1. Most people in the village avoid market food on the whole and make do with what they can grow themselves or buy from trusted neighbors. A subsidy for egg consumption, without addressing the existing constraints on local egg production, would encourage industrial poultry farms while at the same time (through introduced diseases) undermining existing local efforts at self-provisioning locally-produced, organic food.

This example illustrates the complexity of barriers to the provision of sufficient transitional weaning food in one village. It shows that the first necessary intervention would be for the health of chickens. This would require a OneHealth long-term strategy aimed at supporting the villagers to sustain their own efforts to grow their own food (70).

When we ask the question for older children then the 125 children in that age bracket would require an additional 37,500–62,500 cal per day. (Note that at present high protein high fat nutritional supplementation is supplied in plastic sachets from an international aid organization). The answer begins with land, the source of food. These villagers were relocated from a remote mountainous area, where they had laid claim to 8,000 hectares, of which 2,000 could be used for cultivation. Crops of manioc and rice were grown in fields rotated on a 15-years fallow, and fruit and vegetable gardens on a five-to-seven-years fallow rotation. Diets were supplemented with gathering, fishing and hunting in the remaining territories, domestic animals, and trade (63, 71).

The village, then numbering about 900 individuals, relocated to a more accessible area in 1996. New Kandon now lays claim to roughly 800 hectares, divided between upland fields, forests for gathering, wet rice fields and gardens. Originally, 1,222 hectares had been promised by the District, but in the years following their resettlement the original occupants of the area objected and were able to retain control of a significant amount of their original territory, to the detriment of the new settlers. On settlement, the village split the land equally among every individual. Since then, the population has grown but land has not been redistributed. Additionally, in 2006, the District authorities granted 84 hectares of food gardens in Kandon to a Vietnamese rubber company. This occurred without the consent of the settlers: they explained that a neighboring village had agreed to the project and it was presented to New Kandon as a *fait accompli*. The village received an electricity connection in return for the land, and some wage work on the plantation, but people said that they would much rather have the land back for growing food on. They also raised concerns about the chemicals used by the plantation, which is very close to village homes and the school, as well as the village water supply.

There are no spare gardens or fields that could be used to feed these children. This would require people to find money and then access the market to buy food. The food would likely be driven in from elsewhere, and also involve significant carbon in its production. Because people would much rather grow their own food organically, and know where it came from, this monetization and carbonization of their food chain is not a cultural preference. In this index village people are happy to earn cash and enjoy limited consumer goods, they are also keenly interested in the health benefits of growing their own food. This requires access to land so people can grow their own food.

This thought strand leads to a consideration of the implications of the carbon economy with respect to “food miles” (72). If people were incentivized to buy more food from the market then they would be driven further into the cash economy and access food that had been trucked in from elsewhere, and villagers would need to drive to markets to access it.

This vignette from the lived experience of villagers illustrates the close connection and dynamic tension between their biological adaptation to their available food supply, with a final height reduced from its genetic potential, and the coercive force to adapt to a market economy that erodes their access to land, erodes their cultural and nutritional self-sufficiency, and brings with it the inevitable threat of the double burden of malnutrition. Indeed, when people in this village were asked what they saw as the major health problems, they identified, along with TB, rising rates of diabetes and cancer. As one woman said “we never had diabetes in the mountains.”

The story of this village demonstrates that food energy sourced from industrial agriculture has multiple ramified effects. The increased calories and narrowed micronutrients conflict with existing biological adaptation to a quite different nutritional profile. Additionally, there are cultural implications, as industrialized agriculture is not only antithetical to people's interest in organic self-sufficiency, it also undermines efforts at continued self-sufficiency by taking the best land and introducing degradations such as chemical pollution and the disease burden from Influenza virus H5N1.

## Conclusion

The double burden of malnutrition requires a double-duty response (37, 73), but this should extend beyond the factors that comprise the usual frame of reference of public health (e.g., dietary patterns, socio-economic factors, hygiene, and sanitation), and include, for example, an ethnographic approach that identifies the cultural barriers to the provision of sufficient food to babies from 6 months of age (8, 71).

For the double burden of malnutrition to be eradicated the focus should, self-evidently, be on the education, vocational training, and nutrition of adolescent girls in emerging economies so that they have the freedom of choice to delay their first pregnancy until they are socially and physically mature.

The erasure of fetal growth constraint, and post-natal adaptation to an insufficient increase in food energy intake during the transitional period with subsequent growth faltering, is contingent on the multi-level approach. This extends from addressing the politics of structural inequality in societies of increasing wealth disparity to the cultural factors that coerce adolescents into pregnancy, and the washing out of the probable epigenetic factors that requires several generations of optimal nutrition.

However, nutritional justice demands that the well-understood approach to the consequences of metabolic adaptation in the global South are matched by social (group) and structural adaptation in the global North. This requires a planetary health perspective in which group selection of traits that are necessary for adaptation to the known future consequences of climate change, in this case behavioral changes to food sustainability, are at one end of a continuum with at the other end, the structural changes needed to erase the metabolic effects of maladaptation to the rapid shift in diet in an emerging or post-industrial economy.

## Author's Note

The germ of these ideas was planted long ago at the Royal Children's Hospital, Melbourne, by Dr. John Court. He continues to inspire.

## Author Contributions

All authors listed have made a substantial, direct and intellectual contribution to the work, and approved it for publication.

### Conflict of Interest

The authors declare that the research was conducted in the absence of any commercial or financial relationships that could be construed as a potential conflict of interest.
